# The relative reinforcing efficacy of nicotine in an adolescent rat model of attention-deficit hyperactivity disorder

**DOI:** 10.3389/fpsyt.2023.1154773

**Published:** 2023-05-15

**Authors:** John R. Smethells, Danielle Burroughs, Amy Saykao, Mark G. LeSage

**Affiliations:** ^1^Hennepin Healthcare Research Institute, Minneapolis, MN, United States; ^2^Department of Medicine, University of Minnesota Medical School, Minneapolis, MN, United States; ^3^Department of Pharmacology, University of Minnesota, Minneapolis, MN, United States; ^4^Department of Psychology, University of Minnesota, Minneapolis, MN, United States

**Keywords:** attention deficit hyperactivity disorder, spontaneously hypertensive rat, tobacco use disorder, nicotine, self-administration

## Abstract

**Introduction:**

Attention-deficit/hyperactivity disorder (ADHD) is an independent risk factor for tobacco use disorder. Individuals with ADHD are more likely to begin smoking at a younger age, become a daily smoker sooner, smoke more cigarettes per day, and exhibit greater nicotine dependence than individuals without ADHD. It is unclear whether these findings are due to the reinforcing efficacy of nicotine *per se* being greater among individuals with ADHD. The purpose of the present study was to examine this issue using an animal model of ADHD, the spontaneously hypertensive rat (SHR) strain.

**Methods:**

Adolescent SHR and Wistar (control) rats were given access to a typically reinforcing nicotine unit dose (30 μg/kg), a threshold reinforcing nicotine dose (4 μg/kg), or saline under an FR 1 (week 1) and FR 2 (week 2) schedule during 23 h sessions to examine acquisition of self-administration. Behavioral economic demand elasticity was then evaluated at the 30 μg/kg dose through an FR escalation procedure.

**Results:**

At the 30 μg/kg dose, SHR rats exhibited a lower average response rate, lower mean active to inactive lever discrimination ratio, and lower proportion of rats acquiring self-administration compared to control rats. During demand assessment, SHR rats showed no significant difference from Wistars in demand intensity (Q_0_) or elasticity (α; i.e., reinforcing efficacy). In addition, no strain difference in acquisition measures were observed at the 4 μg/kg dose.

**Discussion:**

These findings suggest that the increased risk of tobacco use disorder in adolescents with ADHD may not be attributable to a greater reinforcing efficacy of nicotine, and that other aspects of tobacco smoking (e.g., non-nicotine constituents, sensory factors) may play a more important role. A policy implication of these findings is that a nicotine standard to reduce initiation of tobacco use among adolescents in the general population may also be effective among those with ADHD.

## 1. Introduction

Attention Deficit Hyperactivity Disorder (ADHD) is characterized by a variety of behavioral signs, including inattention, hyperactivity, and impulsivity, and is a comorbid condition associated with substance use disorders (1). For example, it is well-established that ADHD is an independent risk factor for tobacco use disorder, including electronic cigarette use. The prevalence of smoking and e-cigarette use is higher in both adults (41%) and adolescents (19–46%) with ADHD than the general population (26% vs. 10–24% for adults and adolescents, respectively), and prevalence increases directly with the number of symptoms reported (up to over 60%; (2–7)). Individuals with ADHD are more likely to begin smoking at a younger age, become a daily smoker sooner, smoke more cigarettes per day, and exhibit greater nicotine dependence than individuals without ADHD (2, 8, 9).

Because the data supporting the relationship between ADHD and tobacco used disorder come from quasi-experimental studies (e.g., cross-sectional, longitudinal observation), the causality of the relationship and mechanisms mediating it remain unclear. However, it appears that one mechanism may be an increase in the reinforcing efficacy of smoking. In a study using self-administration procedures, adult smokers with ADHD worked harder for cigarette puffs under a progressive-ratio (PR) schedule than those without ADHD and they reported higher levels of withdrawal symptoms (10). These findings are consistent with animal studies showing greater reinforcing efficacy of abused stimulants (e.g., cocaine) in rat models of ADHD (11–13). Together, these findings suggest that the reinforcing efficacy of nicotine, the principle reinforcing constituent in cigarette smoke, may be higher in smokers with ADHD. However, human studies have not examined the effects of nicotine in isolation from the other active constituents in cigarette smoke (e.g., acetaldehyde, minor alkaloids) or the sensory factors associated with tobacco use (e.g., smell, taste). As such, it remains unclear whether the increased motivation to smoke in individuals with ADHD is attributable to greater nicotine reinforcement *per se*.

Animal models can help clarify this issue because they allow precise control over nicotine exposure and design of true experiments to examine how nicotine may influence initiation of tobacco use in adolescents, which is unethical to study in humans. Numerous preclinical studies show that pre-and postnatal nicotine exposure can increase ADHD-related behaviors in rats (e.g., locomotor activity, impulsive action, impulsive choice), suggesting a causal role of nicotine exposure in ADHD (14–18). However, few studies have examined the inverse relationship. Although no animal model can fully recapitulate all neurobehavioral features of ADHD, the Spontaneously Hypertensive Rat (SHR) rat is one of the most common animal models of ADHD that has considerable face, construct, and pharmacological validity (19–23). Thus, nicotine self-administration in SHR rats provides a model to examine relationships between ADHD and nicotine reinforcement. To date, only two studies have examined nicotine self-administration in SHR rats. Chen et al. (24) examined nicotine self-administration (NSA) under a fixed-ratio (FR) 1 schedule in several strains of adolescent male rats. They found that NSA was higher in SHR rats compared to Wistar-Kyoto (WKY) rats (a control strain), but not compared to Lewis rats, a strain often used in NSA research. In contrast, Han et al. (25) found that NSA under an FR 10 schedule did not differ between adolescent female SHR and WKY rats, and both strains showed lower NSA compared to Lewis rats. This study was unusual in that it used a social learning paradigm and licking response with an oral cue to earn nicotine infusions. In addition, these studies used the SHR strain from Harlan Laboratories (SHR/NHsd), which is less well validated compared to the strain from Charles River Laboratories (SHR/NCrl (23)). Moreover, neither study used procedures designed to examine strain differences in motivation or reinforcing efficacy *per se*.

The purpose of the present study was to compare NSA in SHR/NCrl and Wistar control rats using a behavioral economic approach, in which elasticity of demand (i.e., the rate at which consumption declines with increases in price/effort) provides a measure of the motivational or reinforcing strength of nicotine. We used rats from Charles River Laboratories because SHR/NCrl rats display all the core characteristics of ADHD (23). We studied adolescent SHR/NCrl rats because a) adolescents are more vulnerable to substance use disorders, including tobacco use disorder (26), b) almost all smokers begin smoking and become nicotine dependent during adolescence (27, 28), and c) ADHD and tobacco use disorder are comorbid in adolescents (see above). In addition, males and females were included in light of the apparent sex differences in SHR/NHsd rats mentioned above and because sex differences in smoking/nicotine reinforcement and comorbid disorders have been reported in humans and animal models (29–32). However, sex differences were not a primary focus of the present study. An initial pilot study was conducted to confirm phenotype differences (hyperactivity and impulsivity) between strains. Our hypothesis was that the reinforcing efficacy of nicotine would be greater in SHR rats than Wistar controls, as indicated by a higher rate of acquisition and greater behavioral economic demand (i.e., lower elasticity) for nicotine.

## 2. Materials and methods

### 2.1. Animals

Male and female preadolescent SHR/NCrl, Wistar, and Wistar-Kyoto (WKY) (Charles River Laboratories, Chicago, IL) rat pups were shipped to arrive on post-natal day (PND) 25 and were housed with free access to chow and water in a temperature- (22°C) and humidity-controlled colony room until catheter implantation on PND 33. Importantly, rats from Charles River Laboratories were used because SHR rats from this vendor have been validated more extensively than those from other vendors (23). Following catheter implantation, rats were individually housed in an operant chamber and provided free access to water and restricted access to food, beginning at 13 g/day and escalated weekly to 16 g and then 18 g/day, where the food allotment remained for the rest of the protocol. Based on pilot data, this regimen provides an amount of food per gram of body weight comparable to the level of food restriction often used in adult rats. Protocols were approved by the Hennepin Healthcare Research Institute’s Institutional Animal Care and Use Committee and were in accordance with NIH guidelines set forth in the Guide for the Care and Use of Laboratory Animals (National Research Council, 2011).

### 2.2. Apparatus

#### 2.2.1. Open field locomotor arena

A clear plexi-glass sided rectangular box with a solid white PVC composite bottom plate, clear plexiglass walls and an open top (43 cm width × 43 cm depth × 30 cm height; Med Associates, St. Albans, VT) was used for locomotor activity testing. Each box contained a 16 × 16 photocell array 4 cm above the floor to track horizontal movement and a 16 beam array 18 cm from the floor to count rearings. Activity Monitor 5 software was used to measure locomotor activity (Med Associates).

#### 2.2.2. Operant chambers

Nicotine self-administration chambers (Med-Associates, St. Albans, VT) were composed of aluminum and polycarbonate walls and a stainless-steel grid floor. The chamber had two response levers on the front panel, each with a white stimulus light located directly above, and the back panel contained a house light mounted centrally at the top with a waterspout below. Chambers were contained in sound-attenuating boxes equipped with ventilation fans that provided masking noise. Infusion pumps (Model RHSY, Fluid Metering, Syosset, NY) were connected to tygon tubing that attached to a swivel (Instech Inc., Plymouth Meeting, PA) affixed to a counter-balanced arm and centered over the opening in the ceiling of the experimental chamber. Tubing from the swivel ran through a spring leash that attached to a vascular access harness (VAHD115AB, Instech) worn by the rat. Similar chambers were used in the pilot study to examine lever pressing under a differential-reinforcement-of-low-rate (DRL) schedule of food delivery for comparing impulsivity between strains. A computer (OS: Windows 7®) running MED-PC IV® (Med Associates) controlled experimental sessions and recorded data.

### 2.3. Drugs

(−) Nicotine base (Sigma Chemical Co., St. Louis, MO) was dissolved in saline to formulate all nicotine doses (4 & 30 ug/kg; doses expressed as the base). The PH of each solution was adjusted to 7.4 with NaOH and then heparin was added (30 units/mL) to aid catheter patency. The concentration of nicotine in each dilution was verified using gas chromatography with nitrogen phosphorous detection using our routine assay (33).

### 2.4. Procedure

#### 2.4.1. Pilot study of strain differences in locomotor activity and impulsivity

A pilot study was conducted in periadolescent male rats (*N* = 8 per strain) to confirm the expected phenotypes. First, all rats began four daily open field testing sessions beginning on PND 32 to examine differences in locomotor activity between strains. Rats were not habituated to the testing chamber prior to measuring locomotor activity on the first day. Each day, each rat was transported in a shoe box cage to a dimly lit testing room and allowed 1 h to acclimate to the room before testing. Rats were then placed in the open field activity arena for 30 min and beam breaks were recorded to determine the horizontal distance traveled. Impulsivity testing then began on PND 36. During this phase, rats were trained to lever press for 45 mg food pellets on a fixed-ratio (FR) 1 schedule of food delivery during daily 30-min sessions. Once lever pressing was established (at least 50 pellets earned within 60 min), rats were placed on a DRL 1-s schedule of food delivery. Under this schedule, pressing the active lever produced a food pellet only after a delay of 1 s had elapsed since the previous active lever press. Premature presses made on the active lever during the delay reset the delay timer and were considered the measure of impulsivity (i.e., poor inhibitory control). The DRL interval was 1 s for one session, then increased to 3 s for one session, and then to a terminal 5-s for 10 sessions. Sessions ended after 30 min or delivery of 50 pellets, whichever occurred first.

#### 2.4.2. Open field testing prior to nicotine self-administration (NSA)

All rats in the NSA study underwent one open field testing session on PND 32 to compare phenotype differences in locomotor activity between strains observed in the pilot study. Apparatus and procedures were identical those used in the pilot study. Only one locomotor activity session was conducted and impulsivity was not measured in these rats to minimize delaying the start of NSA training and ensure the protocol could be finished within the adolescent period. WKY rats were not used for the self-administration study because they are another inbred strain and considered a model of other human psychiatric disorders (e.g., depression). They are therefore inappropriate as a model of the general population of tobacco users.

#### 2.4.3. Surgical procedures

On PND 33, rats were implanted with a chronic indwelling catheter into the right jugular vein under isoflurane anesthesia. The catheters exited the body between the scapulae and attached to the vascular-access harness. Immediately following surgery, rats were administered extended-release meloxicam (4 mg/kg; s.c.) for analgesia. Rats recovered for 3 days in the operant chamber, during which time they were given daily catheter flushes of heparinized saline (30 units/mL; i.v.) and ceftriaxone (5.25 mg). Infusions of methohexital (50 mg/mL, i.v.) were provided at critical experimental time points (i.e., following FR 1, FR 2, and the end of demand assessment) to confirm catheter patency. If a catheter lost patency, the rat was removed from the study and its data were not included in the analysis.

#### 2.4.4. Acquisition of self-administration and measurement of behavioral economic demand

Following recovery from surgery, periadolescent (PND 37) rats were given access to nicotine during daily 23-h sessions (12:12 light dark cycle; lights off at 1000 h). The start of each session was signaled by the illumination of the house light in the chamber and initiation of a fixed-ratio (FR 1) schedule of nicotine delivery. Under this schedule, a response on the active lever illuminated the stimulus light above the lever and delivered a nicotine infusion (100 μL/kg @ 50 μL/s). Each nicotine delivery was followed by a 7-s post-infusion timeout, wherein responses were recorded but had no programmed consequence. After the timeout, the stimulus light was turned off, and nicotine was again available under the FR 1 schedule. Responses on the inactive lever were recorded but had no programmed consequence. Three groups were assigned to a unit nicotine dose of either 30 μg/kg (Wistar: *N* = 20 [10 male, 10 female]; SHR/NCrl: *N* = 27 [12 male, 15 female]) or 4 μg/kg (Wistar: *N* = 12 [6 male, 6 female]; SHR/NCrl: *N* = 10 [6 male, 4 female]) or saline (Wistar: *N* = 8 [5 male, 3 female]; SHR/NCrl: *N* = 12 [6 male, 6 female]). These nicotine unit doses were chosen because the higher one is a common training dose for NSA, allowing assessment of elasticity of demand, while the lower unit dose is near the threshold reinforcing unit dose for NSA during 23 h access (32, 34–37), which may allow strain differences in sensitivity to nicotine reinforcement to manifest more readily. More rats were used for the higher unit dose to obtain an adequate sample size for assessing behavioral economic demand. Ground food was placed on the active lever for the first session to facilitate contact with the reinforcement contingency. Following 7 additional sessions, the FR requirement was increased to FR 2 for 7 more sessions.

Acquisition criteria for nicotine self-administration were (1) an average ratio of 2:1 active to inactive lever responding across the most recent three consecutive sessions starting at session three and (2) the average nicotine infusions earned over the same set of sessions had to be above the 95% confidence interval of the mean of saline controls across the same three sessions.

Rats that met acquisition criteria at the 30 ug/kg dose then underwent behavioral economic demand assessment after the last FR 2 session during adolescence (PND 52). Some rats failed to meet the active: inactive response criterion, but exhibited considerable nicotine intake. Demand was also measured in these rats and, because they met the active: inactive response criterion during this phase, they were considered to have acquired NSA and their data were included in the demand analysis. Thus, a total of 14 Wistar (Male: *n* = 6; Female: *n* = 8) and 14 SHR/NCrl (Male: *n* = 7; Female: *n* = 7) completed the demand phase. During this phase, the FR requirement was increased daily (per the progression: 3, 6, 9, 15, 30, 60, 120, 240, 480, etc.) until 0 infusions were earned. Elasticity of demand at the 4 μg/kg nicotine dose was not assessed because too few rats acquired self-administration at that dose.

### 2.5. Data analysis

#### 2.5.1. Locomotor activity

Total horizontal distance (cm) and horizontal distance (cm) traveled in 10-min segments of each session were compared between strains using two-way ANOVA using the Geisser–Greenhouse correction (strain and session or segment as factors), followed by Bonferroni multiple comparisons within and between strains. Data were lost for 11 Wistar (7 male, 4 female) and one male SHR/NCrl rat in the NSA study due to a computer hard drive failure that corrupted their data files. Thus, this analysis included 29 Wistar (14 male, 15 female) and 48 SHR/NCrl (23 male, 25 female) rats for the self-administration study.

#### 2.5.2. Impulsivity

Mean premature responses over the last 3 sessions under the terminal DRL schedule were compared between strains using Brown-Forsythe and Welch ANOVA followed by Dunnett T3 multiple comparisons.

#### 2.5.3. Daily patterns of NSA

Strain differences in daily patterns of NSA were examined using two methods. The first was to assess daily strain differences in active and inactive lever responding during acquisition using a separate two-way (Strain × Session) ANOVA for each FR phase at each unit dose with a Bonferroni correction applied to the main effect of strain (*p >* 0.025) at each FR. The second method was to assess the daily proportion of rats that met acquisition criterion across each session of the acquisition phase (sessions 3–14). To analyze strain differences in this measure, Fischer’s Exact test was conducted on the proportion of rats meeting criteria on the final session of each FR, with a Bonferroni correction for the multiple comparisons across FR value (*p >* 0.025). Because no sex differences in these measures were observed, data were pooled across sex for these analyses.

#### 2.5.4. Exponential demand assessment

To quantify demand elasticity across a range of nicotine unit prices at the 30 μg/kg unit dose, exponential demand curves were fit to nicotine consumption in mg/kg at each FR value for both individual subjects and group means using the Hursh and Silberberg demand equation (38):


(1) $$\log\;Q=\log\;Q_{0}+k\left(e^{-\alpha Q_{0}C}-1\right)$$

In this equation, Q is the quantity of a commodity consumed (mg/kg of Nic), C is unit-price cost of the commodity (FR/mg/kg Nic), and Q_0_ and *α* are free parameters resulting from the best-fit function and refer to maximal consumption at zero price (i.e., demand intensity) and rate of change in consumption across price (i.e., demand elasticity), respectively. The parameter, *k,* is a constant fit globally across groups to normalize consumption. This allows for comparisons of free parameter estimates (i.e., *α* and Q_0_) of individual subjects between different demand functions. The parameter α provides a measure of demand elasticity, how rapidly consumption decreases in response to increases in price. This parameter is inversely related to reinforcing efficacy or essential value. Commodities that have larger α values have more *elastic* demand (i.e., rapid decrease in consumption) and less reinforcing efficacy, whereas those with smaller α values have more *inelastic* demand (i.e., slower decrease in consumption) and greater reinforcing efficacy. An Excel template was used to calculate *P*_max_ (price at which consumption becomes relatively elastic) and O_max_ (maximum level of responding) values for each subject using the group fit *k* (2.218) and the individually fit Q_0_ and α values. Data were pooled across sex because no sex difference was observed in these measures. Log transforms were used to normalize the distribution of α values. To provide a complete demand function, 0 consumption at the highest unit price was replaced with 0.01 since 0 is undefined on a log scale and the log of 0.01 (i.e., log 0.01 = −2) is the next lowest log-unit value below the log of 1 infusion (i.e., log 0.03 = −1.52). Additionally, to make group fits of demand functions more representative of individual subjects, 0 infusions (i.e., 0.01) were interpolated for each subject from the point where 0 infusions were earned to the highest unit price achieved by an individual rat. These interpolated data were only used to illustrate group-level demand curves and were not used to determine the individual-subject curve fits or to conduct statistical analyses. The mean of parameter values from curves fit to individual subject data were compared between strains using independent-samples *t*-tests with Welch’s correction.

## 3. Results

### 3.1. Locomotor assessment

**Figure 1:**
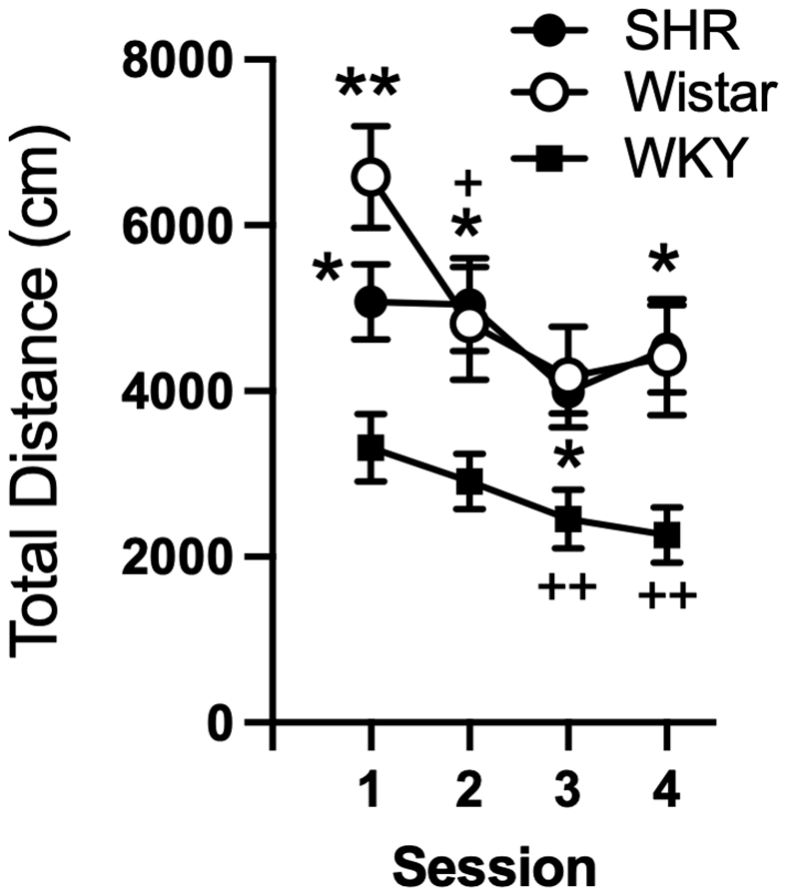
Mean (±SEM) total horizontal distance (cm) traveled during each locomotor activity session in Wistar (open circles), SHR (closed circles), and WKY (closed squares) rats from the pilot study to confirm phenotype differences in locomotor activity between strains. Asterisks indicate significant differences from WKY rats, *p < 0.05, **p < 0.01. Plus symbols indicate significant differences from the first session, ^++^*p* < 0.01, ^+++^*p* < 0.001.

**Figure 2:**
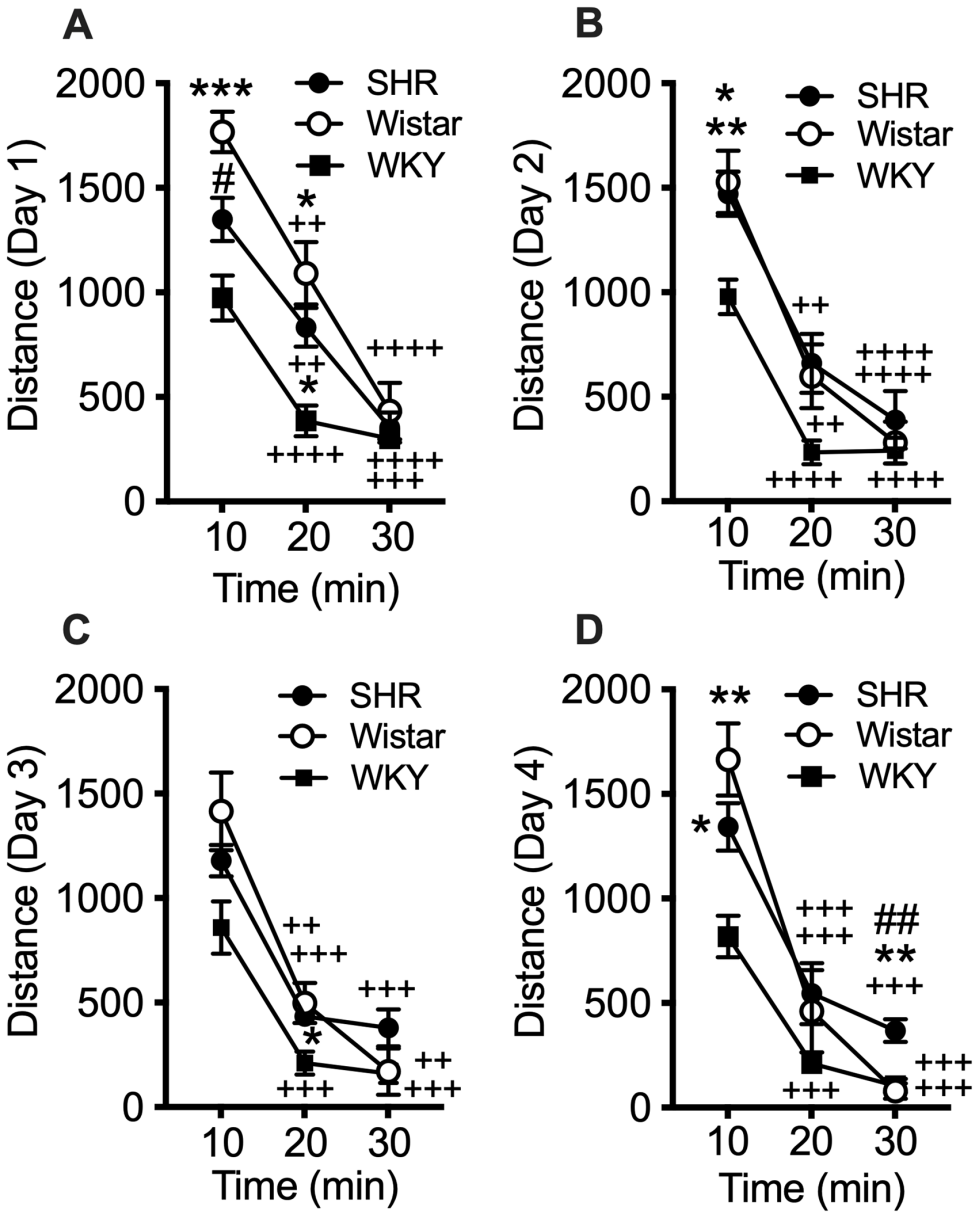
Mean (±SEM) horizontal distance (cm) traveled during consecutive 10 min segments of the locomotor activity sessions in Wistar (open circles), SHR (closed circles), and WKY (closed squares) rats. Each panel shows data from each of four consecutive sessions (**A–D**, respectively; indicated on y-axis) of the pilot study. Asterisks indicate significant differences from WKY rats, *p < 0.05, **p < 0.01, ***p < 0.001. Pound symbols indicate significant differences between SHR and Wistar, ##*p* < 0.01. Plus symbols indicate significant differences from the first 10 min segment, ^++^*p* < 0.01, ^+++^*p* < 0.001, ^++++^*p* < 0.0001.

**Figure 3:**
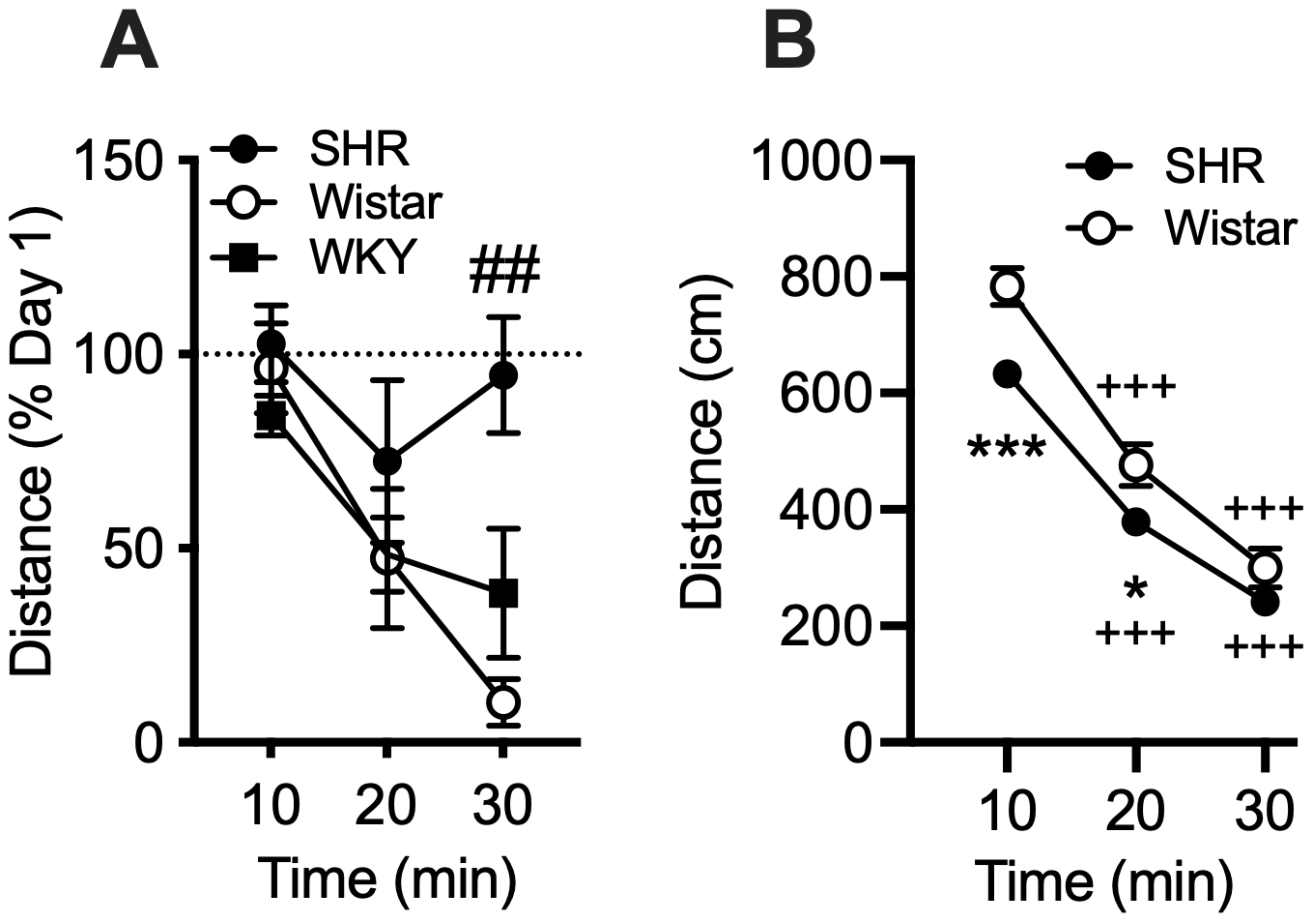
Mean (±SEM) horizontal distance (cm) traveled during consecutive 10 min segments of the locomotor activity sessions in Wistar (open circles), SHR (closed circles), and WKY (closed squares) rats. **(A)** The percent change in distance traveled in each 10 min segment between the first and fourth sessions of the pilot study. Pound symbol indicates significant differencs between SHR and Wistar, ##*p* < 0.01. **(B)** Data from all rats in the self-administration study. WKY rats were not used in the self-administration study. Asterisks indicate significant differences between strains, *p < 0.05, **p < 0.01, ***p < 0.001. Plus symbols indicate significant differences from the first 10 min segment, ^++^*p* < 0.01, ^+++^*p* < 0.001, ^++++^*p* < 0.0001.

Data from the pilot study showed a significant effect of strain (*F*_2, 21_ = 10.21, *p* < 0.001) and session (*F*_3, 58_ = 8.49, *p* < 0.001), but no significant interaction (Figure 1). Total distance traveled per session was significantly higher in SHR rats compared to WKY rats, but not Wistar rats, during every session (Figure 1). Wistar rats only differed from WKY rats on the first session. There were also significant strain differences in the within-session pattern of locomotor activity. There was a significant effect of strain during every session (F_2, 21_ = 10.66, *p* < 0.001; F_2, 21_ = 4.63, *p* < 0.05; *F*_2, 21_ = 4.69, *p* < 0.05; *F*_2, 21_ = 5.46, *p* < 0.05; for sessions 1–4 respectively). There was also a significant effect of time segment during every session (*F*_2, 39_ = 149.3, *p* < 0.0001; *F*_2, 39_ = 117.7, *p* < 0.0001; *F*_2, 39_ = 98.7, *p* < 0.0001; *F*_2, 39_ = 129.9, *p* < 0.0001; for sessions 1–4 respectively). There was a significant strain x time segment interaction during every session, except session two (*F*_4, 42_ = 6.61, *p* < 0.001; *F*_4, 42_ = 2.89, *p* < 0.05; *F*_4, 42_ = 6.81, *p* < 0.001; for sessions 1, 3, and 4 respectively). Overall, SHR rats were more active during the 10 or 20 min segments of the session compared to WKY, but not Wistar, rats during every session (Figure 2). The mean (±SEM) distance traveled was lower in SHR rats compared to Wistars in the first 10 min of the of the first session (*t*_13.9_ = 2.95, *p* < 0.05; Figure 2A), but by the fourth session, distance traveled was significantly higher in SHR rats compared to both Wistars and WKYs in the last 10 min of the session (*t*_12.1_ = 1.57, *p* < 0.01; *t*_13.8_ = 3.49, *p* < 0.05; Figure 2D). In addition, there was no significant change in distance traveled in SHR rats between the first and last session (Figure 3A), whereas Wistar rats showed a significantly greater reduction in distance traveled in the last 10 min of the fourth session (*t*_7.8_ = 5.23, *p* < 0.01). These data demonstrate a hyperactivity phenotype in SHR/NCrl rats from the vendor used for the present study.

Similar to the pilot study, data from rats in the self-administration study showed the mean (± SEM) distance traveled by SHR rats was significantly lower compared to Wistar rats during the initial 10 and 20-min segments of the session (*t*_228_ = 3.96 and 2.58, *p* < 0.001 and 0.05, respectively; Figure 3B). This confirms that SHR/NCrl NSA rats exhibited a phenotype (hypoactivity in a novel environment) similar to those in the pilot study.

### 3.2. Impulsivity

**Figure 4:**
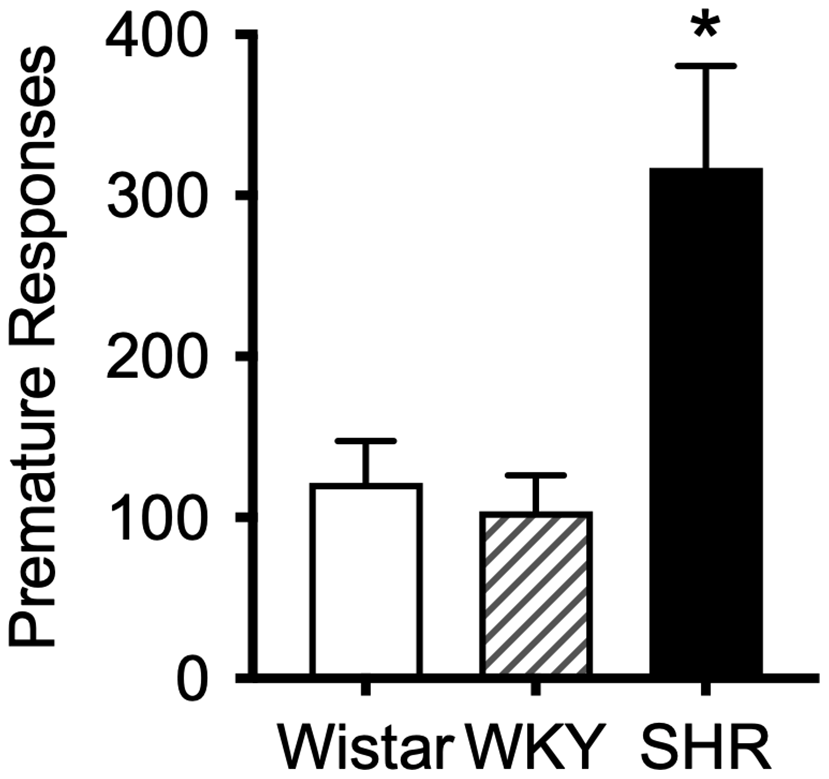
Mean (±SEM) premature responses under a DRL schedule of food delivery in SHR (black bar), Wistar (white bar) and WKY (striped bar) rats. Asterisk indicates significant differences between SHR compared to Wistar and WKY, *p < 0.05.

The mean (±SEM) number of premature responses under the DRL schedule during the pilot study was significantly higher in SHR rats (*F*_8.2, 11.3_ = 8.17, *p* < 0.01) compared to both Wistar and WKY controls (*t*_9.3_ = 2.87, *p* < 0.05; *t*_8.8_ = 3.19, *p* < 0.05; Figure 4). This confirms the presence of the impulsive phenotype in SHR/NCrl rats.

### 3.3. Acquisition of NSA

**Figure 5:**
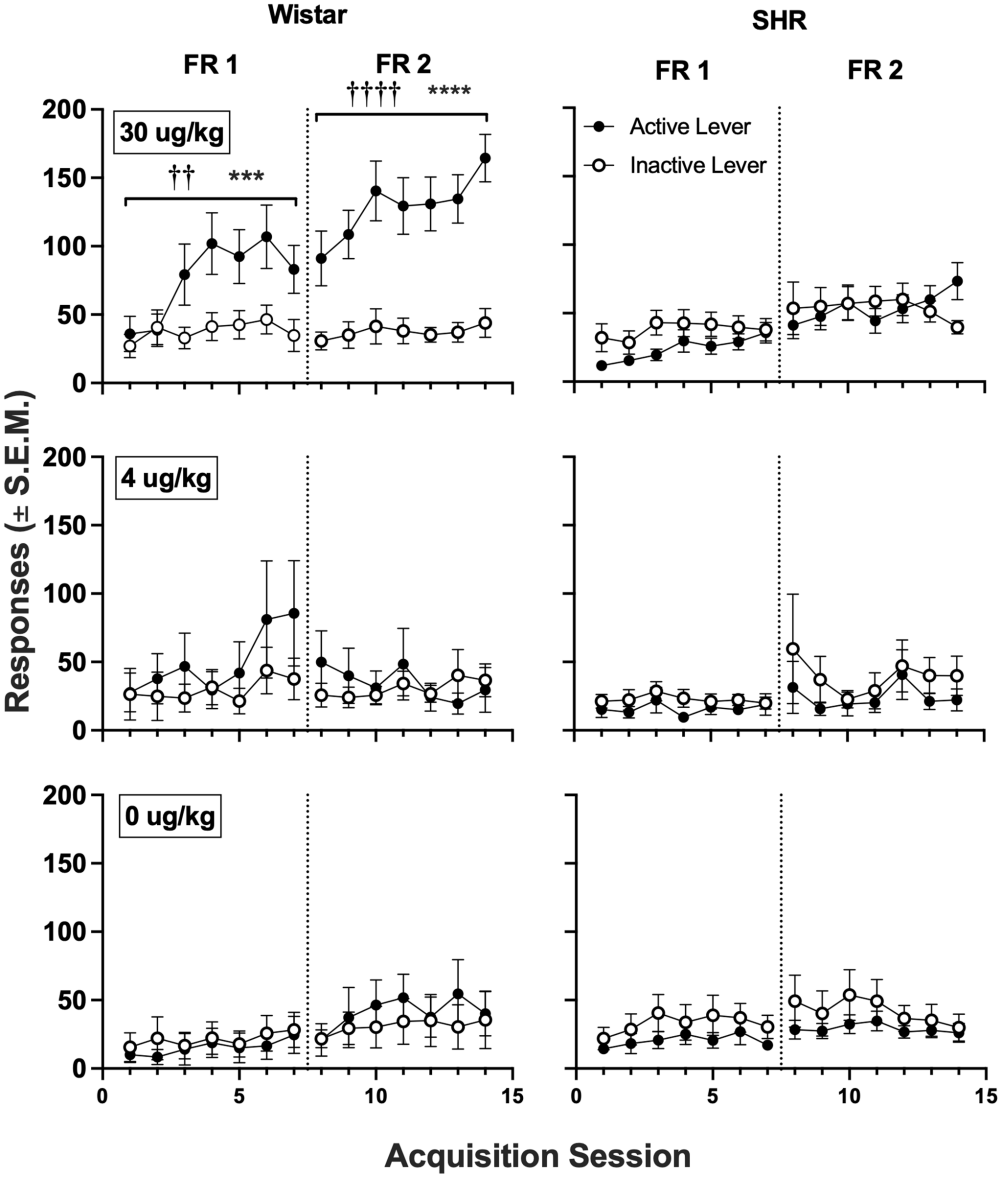
Mean (±SEM) group active and inactive lever responses in all Wistar (left panels) and SHR (right panels) rats across acquisition sessions wherein nicotine was available to self-administer at 30 (top row), 4 (middle row) and 0 μg/kg/infusion of nicotine (i.e., saline; bottom row) under an FR 1 and FR 2 schedule. ^†^Significant main effect of lever, ^††^*p* < 0.01, ^††††^*p* < 0.0001. *Significant main effect of strain, ***p < 0.001, ****p < 0.0001.

Figure 5 shows active and inactive responding during acquisition sessions in Wistar and SHR/NCrl rats across unit nicotine doses. At the 30 μg/kg nicotine dose, only the Wistar rats showed a significant main effect of lever type (i.e., active vs. inactive) on group mean responding, which was observed under both the FR 1 (*F*_1, 19_ = 8.60, *p* < 0.01) and FR 2 (*F*_1, 19_ = 40.82, *p* < 0.001) schedules of reinforcement. Additionally, active lever responding was significantly higher in Wistar rats compared to SHR/NCrl rats during the acquisition phase at the 30 μg/kg dose under both the FR 1 (*F*_1, 45_ = 14.47, *p* < 0.001) and FR 2 (*F*_1, 45_ = 19.47, *p* < 0.0001) schedules. There were no significant main effects of strain or lever type at the 4 or 0 μg/kg doses, indicating rats, on average, did not develop a lever discrimination at these doses.

**Figure 6:**
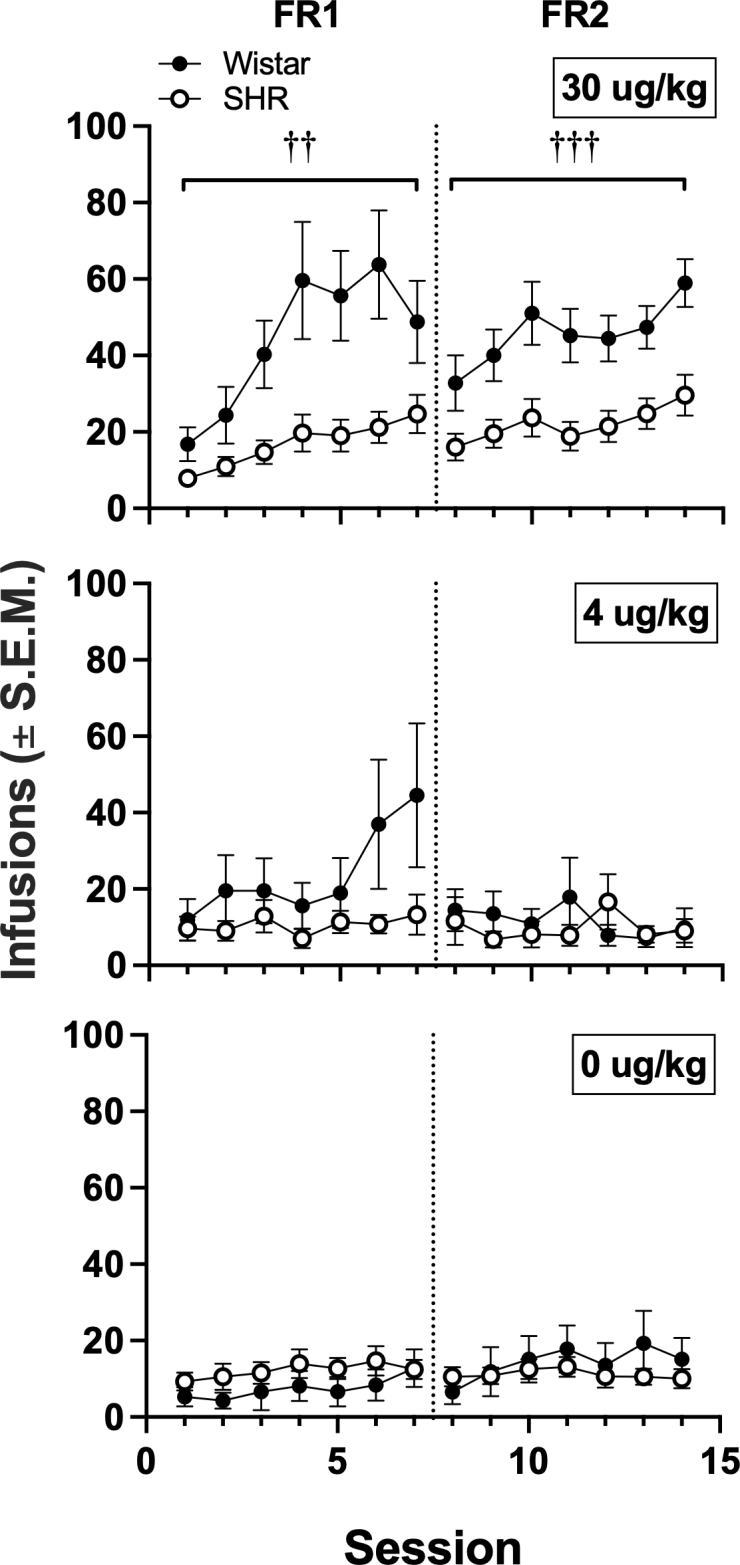
Mean (±SEM) infusions earned in all Wistar and SHR rats across acquisition sessions when self-administering 30 (top row), 4 (middle row) and 0 μg/kg (i.e., saline; bottom row) of nicotine under an FR 1 and FR 2. ^†^Significant main effect of strain, ^††^*p* < 0.01, ^†††^*p* < 0.001.

**Figure 7:**
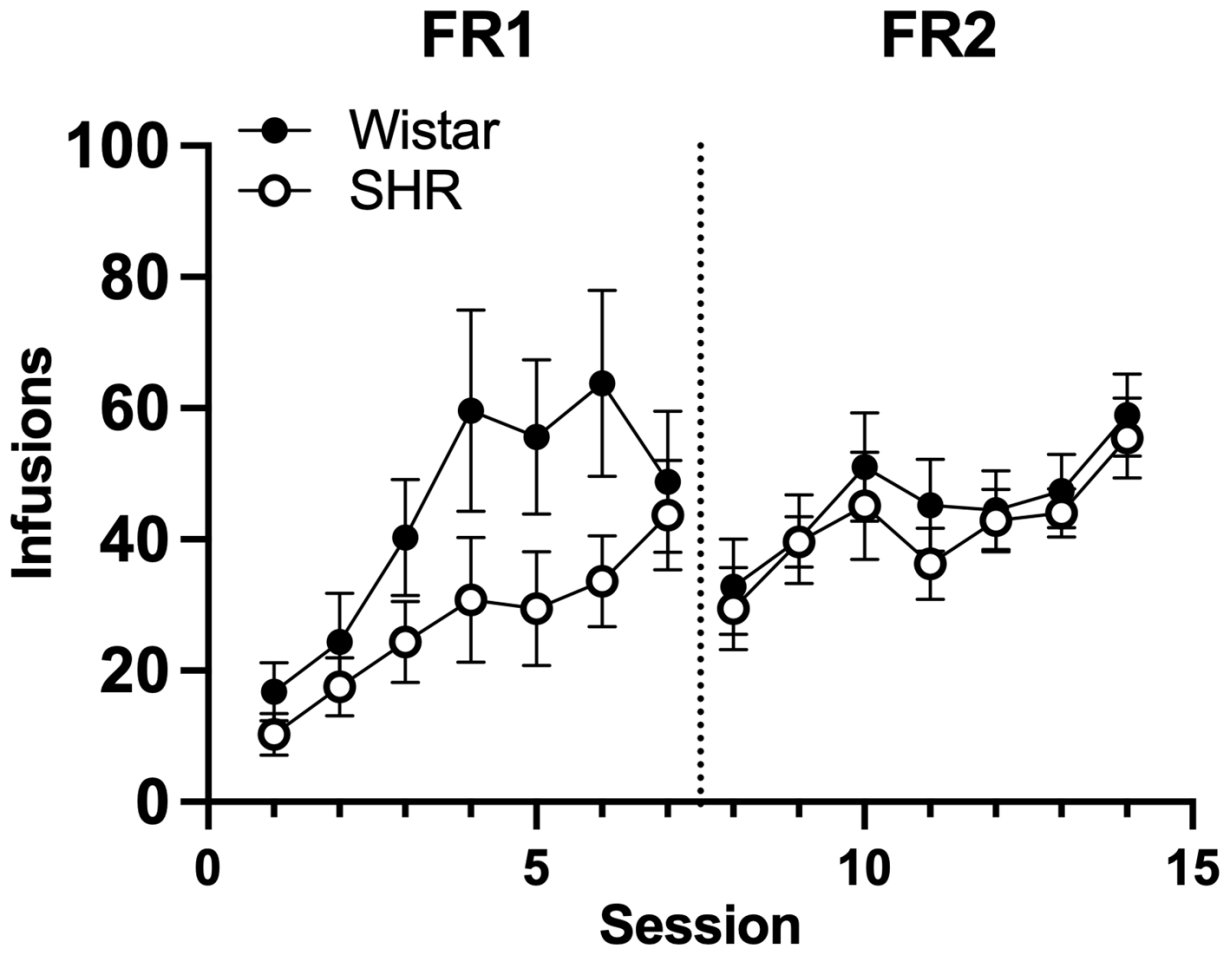
Mean (±SEM) group infusions earned in only Wistar and SHR rats that met acquisition criteria at the 30 μg/kg unit dose.

Figure 6 shows the mean infusions earned by each strain across the three nicotine doses. Wistar rats earned significantly more nicotine infusions than SHR/NCrl rats at the 30 μg/kg nicotine dose under the FR 1 (*F*_1, 45_ = 11.73, *p* < 0.01) and FR 2 (*F*_1, 45_ = 13.94, *p* < 0.001) schedules of reinforcement. No significant strain difference was observed at the 4 or 0 μg/kg doses. Figure 7 shows the mean number of infusions earned in each strain for only those rats that met acquisition criteria. There were no significant differences between strains at either FR schedule.

**Figure 8:**
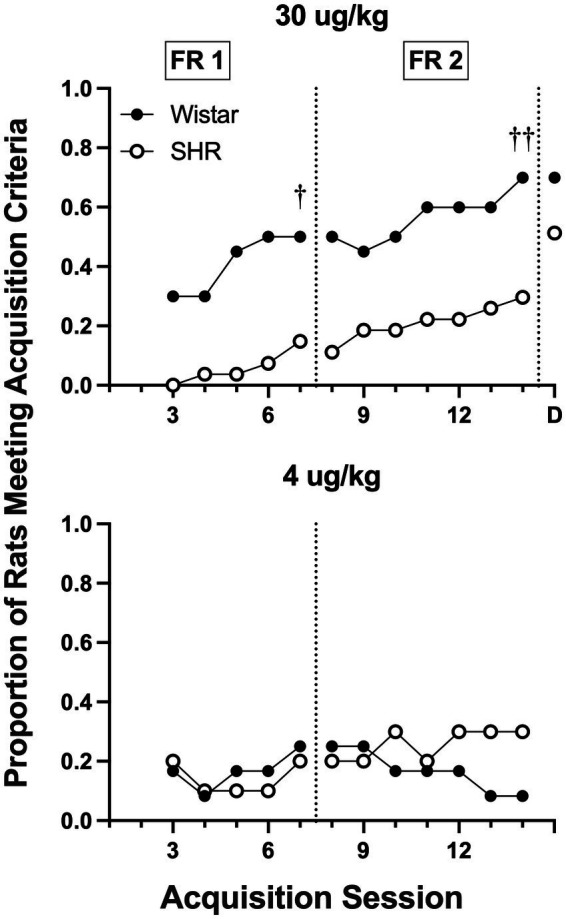
Proportion of rats meeting acquisition criteria at each session of acquisition and during demand assessment (D) in Wistar and SHR rats at the 30 μg/kg (top panel) and 4 ug/kg (bottom panel) doses of nicotine. Significant difference in active lever responses between strains at the end of FR 1 and FR 2, ^†^*p* < 0.05, ^††^*p* < 0.01, respectively.

Figure 8 shows the proportion of rats that met acquisition criteria across acquisition sessions at the 30 and 4 μg/kg doses of nicotine. Wistar rats had a higher proportion meeting the acquisition criteria at the 30 μg/kg dose during both the FR 1 (*p =* 0.012) and a FR 2 (*p =* 0.009) phases of acquisition testing. No strain differences were observed at the 4 μg/kg dose.

### 3.4. Behavioral economic demand

**Figure 9:**
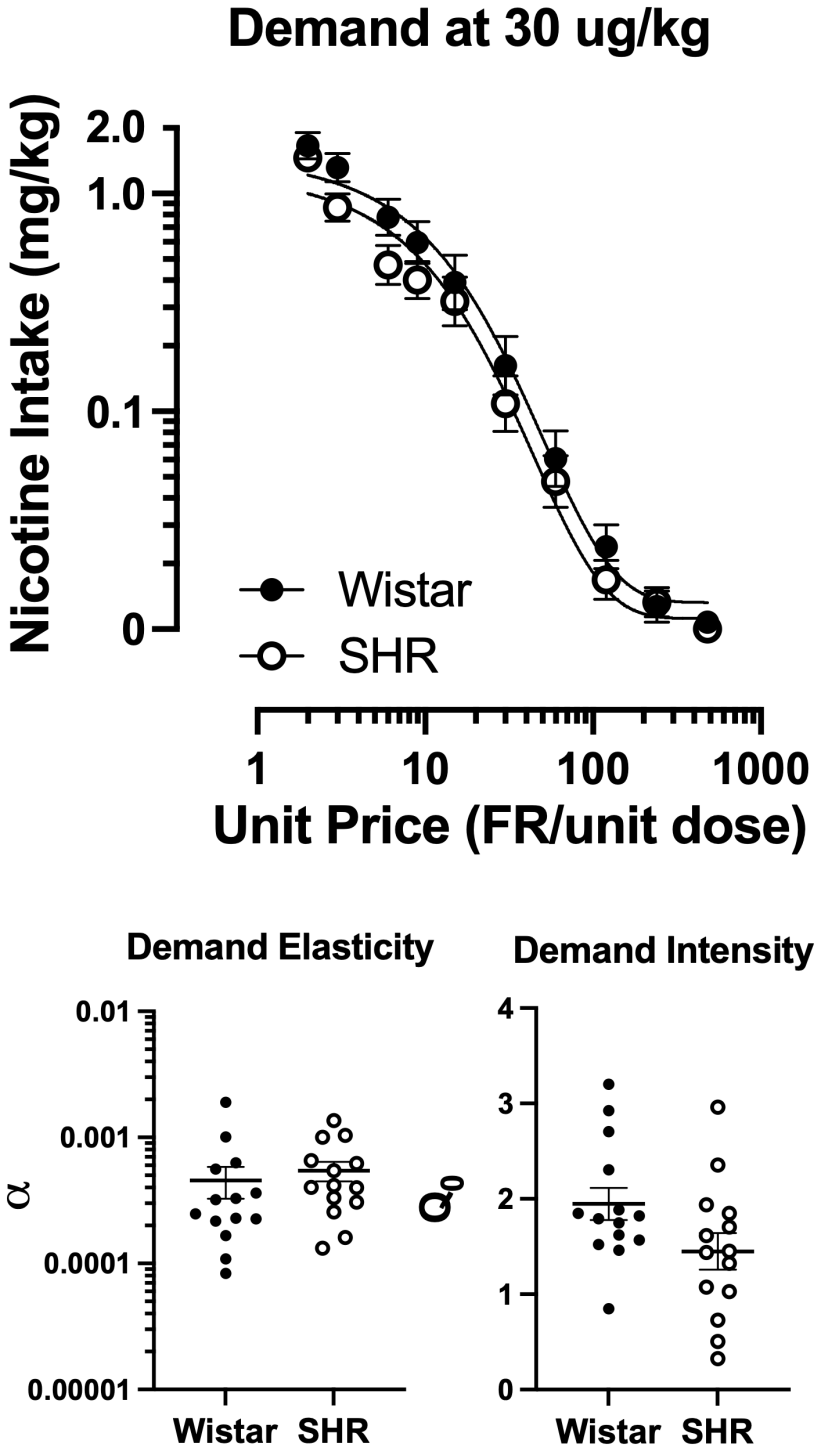
Group mean (±SEM) nicotine intake across unit price in SD and FSL rats during the demand assessment (left panel) at the 30 μg/kg unit nicotine dose and the resulting α values from the individually fit demand functions (right).

Figure 9 shows the mean consumption of nicotine at the 30 μg/kg dose (Top panel) and the best-fit individual demand elasticity (*α*) and demand intensity (Q_0_) values in Wistar and SHR/NCrl rats (Bottom left and right panels, respectively). Table 1 shows individual and mean best-fit exponential demand parameter values. No strain or sex differences in individual best-fit parameters were observed at the 30 μg/kg unit dose.

**Table 1:** Exponential demand curve parameters at the 30 ug/kg dose of nicotine (*k* = 2.218).

Wistar						SHR					
ID #	*α*	*Q*_0_	*P*_max_	*O*_max_	*r* ^2^	ID #	*α*	*Q*_0_	*P*_max_	*O*_max_	*r* ^2^
Male											
1	0.000362	1.85	377.9	223.3	0.94	15	0.001000	1.32	190.7	80.8	0.94
2	0.000226	1.75	638.8	357.5	0.96	16	0.000161	1.45	1078.1	501.1	0.98
3	0.000166	1.79	846.8	485.5	0.97	17	0.000620	1.07	379.0	130.2	0.88
4	0.000247	2.92	349.3	326.8	0.94	18	0.000333	1.85	410.8	242.7	0.98
5	0.001899	0.85	156.6	42.5	0.97	19	0.000400	1.03	612.6	201.9	0.94
6	0.000560	1.57	287.5	144.3	0.90	20	0.000544	0.32	1431.6	148.4	0.64
						21	0.000307	1.94	424.0	263.1	0.99
Mean	0.0005832	1.79	749.4	263.3	0.95	Mean	0.000481	1.29	646.7	224.0	0.91
SEM	0.0002705	0.27	103.4	65.0	0.05	SEM	0.000109	0.19	179.71	56.7	0.05
Female											
7	0.000228	1.46	758.4	354.5	0.96	22	0.000655	0.73	530.0	123.3	0.92
8	0.000084	2.30	1310.5	966.0	0.97	23	0.000401	1.70	369.6	201.5	0.96
9	0.000321	2.70	290.5	251.4	0.97	24	0.000415	1.61	377.0	194.7	0.92
10	0.000109	1.62	1433.9	744.6	0.93	25	0.000256	2.36	418.5	315.3	0.82
11	0.000631	1.52	262.9	128.0	0.99	26	0.000132	1.44	1328.4	612.0	0.97
12	0.001010	3.20	78.2	80.0	0.93	27	0.001036	2.96	82.3	78.0	0.93
13	0.000328	1.82	423.0	246.3	0.96	28	0.001362	0.51	366.0	59.3	0.88
14	0.000217	1.89	615.8	371.6	0.99						
Mean	0.000459	2.06	646.7	392.78	0.96	Mean	0.000608	1.62	496.0	226.3	0.91
SEM	0.000113	0.22	175.3	108.89	0.008	SEM	0.000168	0.32	147.9	72.2	0.02
Overall											
Mean	0.000459	1.94	559.3	337.3	0.96	Mean	0.000545	1.45	571.3	225.2	0.91
SEM	0.000129	0.17	109.5	68.3	0.007	SEM	0.000097	0.19	109.6	42.8	0.02

## 4. Discussion

Contrary to our hypothesis, the main finding of the present study was that the reinforcing efficacy of nicotine was not greater in adolescent SHR/NCrl rats, a rodent model of ADHD, compared to Wistar controls. In some respects, nicotine was a less effective reinforcer in SHR/NCrl rats. As a group, SHR/NCrl rats had lower response rates, fewer infusions earned, and a lower proportion of rats meeting acquisition criteria during the acquisition phase at the 30 μg/kg dose. In other respects, there was no difference in the reinforcing efficacy of nicotine between strains. Acquisition of NSA in SHR/NCrl rats that did meet criteria was no different from Wistar controls, and no strain difference in the proportion acquiring NSA was evident if criteria were met during the demand phase. Moreover, numerous parameters of behavioral economic demand in SHR rats that acquired NSA were similar to Wistar controls. In addition, at the 4 μg/kg dose, which is typically in the range of threshold reinforcing doses in other rat strains (35, 37), neither strain acquired NSA. To the extent that adolescent SHR rats model ADHD in adolescents, the present findings suggest that the greater risk of tobacco use disorder in adolescents with ADHD may not be attributable to a greater reinforcing efficacy of nicotine *per se* in that subpopulation.

The present findings contrast with the epidemiological data that show adolescents with ADHD are at higher risk to develop tobacco use disorder/nicotine dependence (2–4). Specifically, adolescents with ADHD begin smoking and become daily smokers at a younger age, smoke more cigarettes per day, and are more nicotine dependent than individuals without ADHD (2, 8, 9). In addition, prevalence of e-cigarette use is higher among adolescents with ADHD compared to the general population (6, 7). Cross-sectional or longitudinal observational methods have been the primary means to study the relationship between ADHD and smoking. Moreover, to our knowledge, no studies have compared the reinforcing effects of nicotine *per se* between smokers with or without ADHD by manipulating the nicotine content in cigarettes or examining the effects of nicotine alone (e.g., *via* inhaler or i.v. infusion). As such, it is unclear whether the relationship is causal and what factors mediate the relationship. The present study contributes important experimental data to this literature, suggesting the relationship between adolescent ADHD and tobacco use disorder may be mediated by factors other than the direct CNS effects of nicotine, such as non-nicotine constituents (acetaldehyde, MAO inhibitors, minor alkaloids) in cigarette smoke or sensory aspects of smoking (gustatory and olfactory stimuli). Nevertheless, the discrepancy of the present findings with the epidemiological data might also reflect limitations in the validity of nicotine self-administration in SHR rats as a model of ADHD/tobacco use comorbidity. It is possible that the subtype of ADHD that the SHR rat models (i.e., combined hyperactive/impulsive/inattentive) is not representative of the subtype that may be more strongly associated with tobacco use disorder in humans. To our knowledge, the relative strength of the association between tobacco use disorder and different subtypes of ADHD has not been well established.

There are discrepancies between the present findings and those from two prior studies in adolescent SHR/NHsd rats showing a) higher NSA rates compared to WKY controls (Wistar controls were not used) in males but not females, b) no difference in NSA compared to Lewis rats males, and c) lower NSA rates compared to Lewis rats in females (24, 25). This discrepancy may be due to numerous procedural differences, such as food training prior to drug availability, social learning procedures, response topography and reinforcement schedule (FR 1 for lever pressing versus FR10 for a licking response), and type of drug-paired stimulus (visual versus oral). Perhaps more important, both prior studies used a different sub-strain of rat, the SHR/NHsd. Although genome sequencing studies show high genetic correspondence and comparable elevations in blood pressure between the SHR/NCrl and SHR/NHsd, the latter are less consistent in expressing an ADHD phenotype and less responsive to ADHD medications (23). Studies that directly compared the behavioral phenotype of these strains showed that SHR/NCrl rats displayed greater impulsive-and compulsive-like behavior than SHR/NHsd. These findings suggest that the sub-strain differences between studies may not be due to differences in the behavioral procedures that were used (39).

The present findings also differ from previous preclinical studies showing greater reinforcing effects of other stimulants in SHR/NCrl rats (20, 23). For example, SHR/NCrl rats exhibit faster acquisition of cocaine self-administration, an upward shift in the ascending limb of the cocaine self-administration dose–response curve, greater reinforcing efficacy (i.e., higher breaking points) under a progressive ratio schedule of cocaine self-administration, and greater cue-induced reinstatement of cocaine self-administration (23). These studies occurred during adulthood, suggesting that age differences in brain development may be one reason for discrepancies with the present study. Moreover, some sub-strains of SHR rat show reduced expression and function of high-affinity nicotinic acetylcholine (nACh) receptors in numerous subregions of cortical, striatal, and thalamic brain areas (40–44). They also exhibit reduced nACh receptor upregulation in response to nicotine exposure (45). These findings suggest that the reduced proportion of SHR rats acquiring NSA in the present study may be due to weaker nicotine-induced activation of mesolimbic dopamine neurons, which mediate nicotine’s reinforcing effects (46–48). In light of the marked individual differences in NSA among SHR/NCrl rats in the present study, future studies should examine whether individual differences in acetylcholinergic and dopaminergic function are correlated with individual differences in acquisition of NSA in SHR/NCrl rats. However, the similarities in NSA observed between SHR/NCrl rats that acquired NSA and Wistar controls (Figures 5, 7) suggest that the difference in acquisition between strains may be due to other mechanisms.

The present findings have important implications for tobacco regulation. The FDA is considering setting a standard for the maximum nicotine level allowed in certain tobacco products (e.g., cigarettes). The goal is to set the standard below the threshold reinforcing level of nicotine in cigarettes to reduce smoking in current users and prevent adolescents from becoming regular users (46, 47). Setting a nicotine standard requires understanding the extent of population variability in the reinforcing effects of nicotine and tobacco products. As such, the FDA needs data from vulnerable subpopulations in which the reinforcing effects of nicotine may be higher than that of the general population, such as adolescents and those with psychiatric comorbidities (48). To the extent that the SHR/NCrl rat is a valid animal model of ADHD, the present findings of reduced acquisition of nicotine self-administration at the 30 μg/kg dose in SHR/NCrl rats and lack of strain differences at a lower near-threshold reinforcing dose of 4 μg/kg suggests that a nicotine standard that reduces initiation of tobacco use among adolescents in the general population may also be effective at doing so in those with ADHD. Human studies will be critical to examine this issue further to determine the generality of the present findings, and because tobacco regulatory policies cannot solely rely on animal data (37, 46).

The present study has several limitations. First, the ADHD phenotype was not fully characterized in rats that underwent NSA, and the hypoactivity that they exhibited is an apparent contradiction to the expected phenotype in SHR/NCrl rats. However, it would not have been possible to measure hyperactivity (*via* repeated testing) and impulsivity and complete the NSA protocol within the adolescent period. Regardless, the pilot study demonstrated that SHR/NCrl rats from the present vendor exhibit the expected hyperactive and impulsive phenotype. The hyperactivity compared to WKY rats was clear. The hypoactivity compared to Wistar controls was transient, with late-session hyperactivity developing gradually over sessions. Other studies have also shown similar strain differences in hyperactivity, with SHR rats consistently being hyperactive in comparison to WKY rats (the most commonly used control strain), but initially hypoactive or similarly active compared to Wistar rats (49–52). It is also important to note that some studies show that children with ADHD may not initially exhibit hyperactivity in novel environments and that hyperactivity can manifest gradually over repeated obeservations (19, 53–55). As such, the consistency of findings from the pilot study with the general literature on the SHR strain and with rats undergoing NSA in the present study supports the notion that the SHR/NCrl rats used for this study provided a model of ADHD.

Second, a detailed dose–response curve was not determined to better characterize strain differences in the nicotine reinforcement threshold. The low proportion of rats acquiring at the 30 μg/kg unit dose suggests that the nicotine reinforcement threshold is considerably higher for SHR/NCrl rats compared to Wistars, as well as other strains used in previous studies (32, 34, 56, 57). From a tobacco regulatory science perspective, it would be useful to have more detailed dose–response studies of SHR/NCrl and Wistar rats to broaden preclinical risk assessment analyses to estimate the population impact of nicotine reduction policies.

Third adult SHR/NCrl rats were not studied. The hypocholinergic function in SHR/NCrl rats discussed above has been shown to increase with age (41, 42). Thus, to the extent that strain differences in the present study are attributable to hypocholinergic function, there could be more marked strain differences in adults. In addition, studying the reinforcement threshold and behavioral economic demand for nicotine for maintenance, rather than acquisition, of NSA in adult SHR/NCrl rats would help address the regulatory question of whether well-established smoking in adults with ADHD would be more resistant to change than the general population as the nicotine content in tobacco products decreases.

Fourth, use of an external silicone strap harness for the catheter preparation in the present study necessitated individually housing rats after weaning, as group housing quickly results in cagemates chewing off the harnesses. Studies have shown that social isolation after weaning has significant behavioral and neurobiological effects (58). As such, the present findings may represent an interaction of strain and social isolation effects. Future studies should avoid this issue by using other catheter preparations (e.g., subcutaneous button with protective metal cap).

Fifth, strain differences in degree of nicotine dependence *per se* were not studied. Because smokers with ADHD exhibit greater dependence (i.e., severity of withdrawal) than those without ADHD, examining SHR/NCrl in models of nicotine withdrawal would be important to examine further the validity of nicotine exposure in SHR/NCrl rats as a model of comorbid tobacco use disorder and ADHD.

Finally, although some studies have shown that the association between ADHD and tobacco use is independent of other psychiatric disorders (59), the association is significantly increased when certain comorbid disorders are present, such as conduct disorder (CD) (60). Future studies of nicotine reinforcement in animal models of CD alone and combined with models of ADHD may be useful for examining the relative causal contribution of these disorders to the development of tobacco use disorder and their neuropharmacological mechanisms.

Despite these limitations, to our knowledge, this is the first study to directly compare the reinforcing efficacy of nicotine between SHR/NCrl and a control strain using a behavioral economic approach. It is also one of only a few to examine any behavioral effect of nicotine in SHR rats. There is relatively little animal research that has explicitly modeled the effects of smoking comorbidities on nicotine reinforcement, beyond polysubstance abuse and comorbid metabolic disorders (e.g., alcohol, diabetes, obesity (61–64)). As such, our findings are an important extension of the literature on the SHR rat as a model of ADHD generally and on the comorbidity of tobacco use disorder and ADHD in particular.

## Data availability statement

The raw data supporting the conclusions of this article will be made available by the authors, without undue reservation.

## Ethics statement

The animal study was reviewed and approved by Institutional Animal Care and Use Committee, Hennepin Healthcare Research Institute.

## Author contributions

ML conceptualized the study and experimental design. ML, JS, and DB finalized the experimental protocols. DB and AS were responsible for daily conduct of the study and data collection. JS and ML supervised conduct of the study, analyzed the data, and drafted the manuscript. All authors contributed to the article and approved the submitted version.

## Funding

This study was supported by NIDA grant R01-DA042525 (ML, Primary Investigator) and Career Development Awards (ML and JS) from the Hennepin Healthcare Research Institute (formerly Minneapolis Medical Research Foundation). These funding institutions had no role in study design, data collection and analysis, or the decision to submit the manuscript for publication.

## Conflict of interest

The authors declare that the research was conducted in the absence of any commercial or financial relationships that could be construed as a potential conflict of interest.

## Publisher’s note

All claims expressed in this article are solely those of the authors and do not necessarily represent those of their affiliated organizations, or those of the publisher, the editors and the reviewers. Any product that may be evaluated in this article, or claim that may be made by its manufacturer, is not guaranteed or endorsed by the publisher.

## References

[1] Rohner H Gaspar N Philipsen A Schulze M. Prevalence of attention deficit hyperactivity disorder (ADHD) among substance use disorder (SUD) populations: Meta-analysis. Int J Environ Res Pu. (2023) 20:1275. doi: 10.3390/ijerph20021275, PMID: 36674031 PMC9859173

[2] Kollins SH McClernon FJ Fuemmeler BF. Association between smoking and attention-deficit/hyperactivity disorder symptoms in a population-based sample of young adults. Arch Gen Psychiatry. (2005) 62:1142–7. doi: 10.1001/archpsyc.62.10.1142, PMID: 16203959

[3] Mihailescu S Drucker-Colín R. Nicotine, brain nicotinic receptors, and neuropsychiatric disorders. Arch Med Res. (2000) 31:131–44. doi: 10.1016/S0188-4409(99)00087-9 10880717

[4] Molina BSG Pelham WE. Childhood predictors of adolescent substance use in a longitudinal study of children with ADHD. J Abnorm Psychol. (2003) 112:497–507. doi: 10.1037/0021-843x.112.3.497, PMID: 12943028

[5] Pomerleau OF Downey KK Stelson FW Pomerleau CS. Cigarette smoking in adult patients diagnosed with attention deficit hyperactivity disorder. J Subst Abus. (1995) 7:373–8. doi: 10.1016/0899-3289(95)90030-6, PMID: 8749796

[6] Xu G Snetselaar LG Strathearn L Ryckman K Nothwehr F Torner J. Association of Attention-Deficit/hyperactivity disorder with E-cigarette use. Am J Prev Med. (2021) 60:488–96. doi: 10.1016/j.amepre.2020.11.010, PMID: 33745521

[7] Kaplan B Marcell AV Kaplan T Cohen JE. Association between e-cigarette use and parents’ report of attention deficit hyperactivity disorder among US youth. Tob Induc Dis. (2021) 19:1–7. doi: 10.18332/tid/136031, PMID: 34140843 PMC8176894

[8] Milberger S Biederman J Farone SV Chen L Jones J. ADHD is associated with early initiation of cigarette smoking in children and adolescents. J Am Acad Child Adolesc Psychiatry. (1997) 36:37–44. doi: 10.1097/00004583-199701000-00015, PMID: 9000779

[9] Rohde P Kahler C Lewinsohn P Brown R. Psychiatric disorders, familial factors, and cigarette smoking: II. Associations with progression to daily smoking. Nicotine Tob Res. (2004) 6:119–32. doi: 10.1080/14622200310001656948, PMID: 14982696

[10] Kollins SH English JS Roley ME O’Brien B Blair J Lane SD. Effects of smoking abstinence on smoking-reinforced responding, withdrawal, and cognition in adults with and without attention deficit hyperactivity disorder. Psychopharmacology. (2012) 227:19–30. doi: 10.1007/s00213-012-2937-0 23247366 PMC3624067

[11] Harvey RC Sen S Deaciuc A Dwoskin LP Kantak KM. Methylphenidate treatment in adolescent rats with an attention deficit/hyperactivity disorder phenotype: cocaine addiction vulnerability and dopamine transporter function. Neuropsychopharmacology. (2011) 36:837–47. doi: 10.1038/npp.2010.223, PMID: 21150910 PMC3055734

[12] Marusich JA McCuddy WT Beckmann JS Gipson CD Bardo MT. Strain differences in self-administration of methylphenidate and sucrose pellets in a rat model of ADHD. Behav Pharmacol. (2011) 22:794–804. doi: 10.1097/FBP.0b013e32834d623e, PMID: 22015805 PMC3381430

[13] Somkuwar SS Jordan CJ Kantak KM Dwoskin LP. Adolescent atomoxetine treatment in a rodent model of ADHD: effects on cocaine self-administration and dopamine transporters in Frontostriatal regions. Neuropsychopharmacology. (2013) 38:2588–97. doi: 10.1038/npp.2013.163, PMID: 23822950 PMC3828528

[14] Kirshenbaum AP Jackson ER Brown SJ Fuchs JR Miltner BC Doughty AH. Nicotine-induced impulsive action. Behav Pharmacol. (2011) 22:207–21. doi: 10.1097/fbp.0b013e328345ca1c, PMID: 21448062 PMC3151674

[15] Dallery J Locey ML. Effects of acute and chronic nicotine on impulsive choice in rats. Behav Pharmacol. (2005) 16:15–23. doi: 10.1097/00008877-200502000-00002, PMID: 15706134

[16] LeSage MG Gustaf E Dufek MB Pentel PR. Effects of maternal intravenous nicotine administration on locomotor behavior in pre-weanling rats. Pharmacol Biochem Behav. (2006) 85:575–83. doi: 10.1016/j.pbb.2006.10.012, PMID: 17141848 PMC1820587

[17] Benwell MEM Balfour DJK. The effects of acute and repeated nicotine treatment on nucleus accumbens dopamine and locomotor activity. Brit J Pharmacol. (1992) 105:849–56. doi: 10.1111/j.1476-5381.1992.tb09067.x, PMID: 1504716 PMC1908718

[18] Íbias J Nazarian A. Sex differences in nicotine-induced impulsivity and its reversal with bupropion in rats. J Psychopharmacol. (2020) 34:1382–92. doi: 10.1177/0269881120937543, PMID: 32684065 PMC7708527

[19] Sagvolden T Russell VA Aase H Johansen EB Farshbaf M. Rodent models of attention-deficit/hyperactivity disorder. Biol Psychiatry. (2005) 57:1239–47. doi: 10.1016/j.biopsych.2005.02.002 15949994

[20] Vendruscolo L Izidio G Takahashi R. Drug reinforcement in a rat model of attention deficit/hyperactivity disorder–the spontaneously hypertensive rat (SHR). Curr Drug Abus Rev. (2009) 2:177–83. doi: 10.2174/1874473710902020177 19630747

[21] Kantak KM Singh T Kerstetter KA Dembro KA Mutebi MM Harvey RC. Advancing the spontaneous hypertensive rat model of attention deficit/hyperactivity disorder. Behav Neurosci. (2008) 122:340–57. doi: 10.1037/0735-7044.122.2.340, PMID: 18410173

[22] Regan SL Williams MT Vorhees CV. Review of rodent models of attention deficit hyperactivity disorder. Neurosci Biobehav Rev. (2022) 132:621–37. doi: 10.1016/j.neubiorev.2021.11.041, PMID: 34848247 PMC8816876

[23] Kantak KM. Rodent models of attention-deficit hyperactivity disorder: an updated framework for model validation and therapeutic drug discovery. Pharmacol Biochem Be. (2022) 216:173378. doi: 10.1016/j.pbb.2022.173378, PMID: 35367465

[24] Chen H Hiler KA Tolley EA Matta SG Sharp BM. Genetic factors control nicotine self-Administration in Isogenic Adolescent rat Strains. PLoS One. (2012) 7:e44234. doi: 10.1371/journal.pone.0044234.t003, PMID: 22937166 PMC3429443

[25] Han W Wang T Chen H. Social learning promotes nicotine self-administration by facilitating the extinction of conditioned aversion in isogenic strains of rats. Sci Rep. (2017) 7:8052–10. doi: 10.1038/s41598-017-08291-5, PMID: 28808247 PMC5556091

[26] Adriani W Laviola G. Windows of vulnerability to psychopathology and therapeutic strategy in the adolescent rodent model. Behav Pharmacol. (2004) 15:341–52. doi: 10.1097/00008877-200409000-00005, PMID: 15343057

[27] Difranza JR Savageau JA Rigotti NA Fletcher K Ockene JK McNeill AD. Development of symptoms of tobacco dependence in youths: 30 month follow up data from the DANDY study. Tob Control. (2002) 11:228–35. doi: 10.1136/tc.11.3.228, PMID: 12198274 PMC1759001

[28] Oloughlin J Difranza J Tyndale RF Meshefedjian G Mcmillan-Davey E Clarke P. Nicotine-dependence symptoms are associated with smoking frequency in adolescents. Am J Prev Med. (2003) 25:219–25. doi: 10.1016/S0749-3797(03)00198-3 14507528

[29] Fattore L Altea S Fratta W. Sex differences in drug addiction: a review of animal and human studies. Women’s Health (Lond Engl). (2008) 4:51–65. doi: 10.2217/17455057.4.1.51 19072451

[30] Perkins KA. Sex differences in nicotine reinforcement and reward: influences on the persistence of tobacco smoking. Nebr Symp Motiv. (2009) 55:143–69. doi: 10.1007/978-0-387-78748-0, PMID: 19013943

[31] Sanchez V Moore CF Brunzell DH Lynch WJ. Sex differences in the effect of wheel running on subsequent nicotine-seeking in a rat adolescent-onset self-administration model. Psychopharmacology. (2013) 231:1753–62. doi: 10.1007/s00213-013-3359-3, PMID: 24271035 PMC3969388

[32] Grebenstein P Burroughs D Zhang Y LeSage MG. Sex differences in nicotine self-administration in rats during progressive unit dose reduction: implications for nicotine regulation policy. Pharmacol Biochem Behav. (2013) 114–115:70–81. doi: 10.1016/j.pbb.2013.10.020, PMID: 24201048 PMC3903094

[33] LeSage MG Keyler DE Collins G Pentel PR. Effects of continuous nicotine infusion on nicotine self-administration in rats: relationship between continuously infused and self-administered nicotine doses and serum concentrations. Psychopharmacology. (2003) 170:278–86. doi: 10.1007/s00213-003-1539-2, PMID: 12898121

[34] Grebenstein PE Burroughs D Roiko SA Pentel PR LeSage MG. Predictors of the nicotine reinforcement threshold, compensation, and elasticity of demand in a rodent model of nicotine reduction policy. Drug Alcohol Depend. (2015) 151:181–93. doi: 10.1016/j.drugalcdep.2015.03.030, PMID: 25891231 PMC4447604

[35] Smith TT Rupprecht LE Denlinger-Apte RL Weeks JJ Panas RS Donny EC. Animal research on nicotine reduction: current evidence and research gaps. Nicotine Tob Res. (2017) 19:1005–15. doi: 10.1093/ntr/ntx077, PMID: 28379511 PMC5896531

[36] Smethells JR Burroughs D Saykao A Pentel PR Rezvani AH LeSage MG. The reinforcement threshold and elasticity of demand for nicotine in an adolescent rat model of depression. Drug Alcohol Depen. (2021) 219:108433. doi: 10.1016/j.drugalcdep.2020.108433, PMID: 33310485 PMC7855441

[37] Donny EC Taylor TG LeSage MG Me L Buffalari DM Joel D. Impact of tobacco regulation on animal research: new perspectives and opportunities. Nicotine Tob Res. (2012) 14:1319–38. doi: 10.1093/ntr/nts162, PMID: 22949581 PMC3611983

[38] Hursh SR Silberberg A. Economic demand and essential value. Psychol Rev. (2008) 115:186–98. doi: 10.1037/0033-295x.115.1.186 18211190

[39] Kantak KM Stots C Mathieson E Bryant CD. Spontaneously hypertensive rat substrains show differences in model traits for addiction risk and cocaine self-administration: implications for a novel rat reduced complexity cross. Behav Brain Res. (2021) 411:113406. doi: 10.1016/j.bbr.2021.113406, PMID: 34097899 PMC8265396

[40] Gattu M Pauly JR Boss KL Summers JB Buccafusco JJ. Cognitive impairment in spontaneously hypertensive rats: role of central nicotinic receptors. I Brain Res. (1997) 771:89–103. doi: 10.1016/s0006-8993(97)00793-2, PMID: 9383012

[41] Gattu M Terry AV Pauly JR Buccafusco JJ. Cognitive impairment in spontaneously hypertensive rats: role of central nicotinic receptors. Part II. Brain Res. (1997) 771:104–14. doi: 10.1016/S0006-8993(97)00794-4, PMID: 9383013

[42] Jr AVT Hernandez CM Buccafusco JJ Gattu M. Deficits in spatial learning and nicotinic–acetylcholine receptors in older, spontaneously hypertensive rats. Neuroscience. (2000) 101:357–68. doi: 10.1016/s0306-4522(00)00377-8, PMID: 11074159

[43] Yamada S Kagawa Y Ushijima H Takayanagi N Tomita T Hayashi E. Brain nicotinic cholinoceptor binding in spontaneous hypertension. Brain Res. (1987) 410:212–8. doi: 10.1016/0006-8993(87)90318-0, PMID: 3594235

[44] Wigestrand MB Mineur YS Heath CJ Fonnum F Picciotto MR Walaas SI. Decreased α4β2 nicotinic receptor number in the absence of mRNA changes suggests post-transcriptional regulation in the spontaneously hypertensive rat model of ADHD. J Neurochem. (2011) 119:240–50. doi: 10.1111/j.1471-4159.2011.07415.x, PMID: 21824140 PMC3171636

[45] Hohnadel EJ Hernandez CM Gearhart DA Terry AV. Effect of repeated nicotine exposure on high-affinity nicotinic acetylcholine receptor density in spontaneously hypertensive rats. Neurosci Lett. (2005) 382:158–63. doi: 10.1016/j.neulet.2005.03.011, PMID: 15911141

[46] Sofuoglu M LeSage MG. The reinforcement threshold for nicotine as a target for tobacco control. Drug Alcohol Depend. (2012) 125:1–7. doi: 10.1016/j.drugalcdep.2012.04.023, PMID: 22622242 PMC3419325

[47] DHHS, FDA. Tobacco product standard for nicotine level of combusted cigarettes. Fed Regist. (2018) 83:11818–43. Available at: https://www.regulations.gov/document?D=FDA-2017-N-6189-0001

[48] Hatsukami DK Benowitz NL Donny E Henningfield J Zeller M. Nicotine reduction: strategic research plan. Nicotine Tob Res. (2013) 15:1003–13. doi: 10.1093/ntr/nts214, PMID: 23100460 PMC3646645

[49] Sagvolden T Hendley ED Knardahl S. Behavior of hypertensive and hyperactive rat strains: hyperactivity is not unitarily determined. Physiol Behav. (1992) 52:49–57. doi: 10.1016/0031-9384(92)90432-2 1529013

[50] Sagvolden T Pettersen MB Larsen MC. Spontaneously hypertensive rats (SHR) as a putative animal model of childhood hyperkinesis: SHR behavior compared to four other rat strains. Physiol Behav. (1993) 54:1047–55. doi: 10.1016/0031-9384(93)90323-8, PMID: 8295939

[51] Sagvolden T Johansen EB Wøien G Walaas SI Storm-Mathisen J Bergersen LH. The spontaneously hypertensive rat model of ADHD–the importance of selecting the appropriate reference strain. Neuropharmacology. (2009) 57:619–26. doi: 10.1016/j.neuropharm.2009.08.004, PMID: 19698722 PMC2783904

[52] Somkuwar SS Kantak KM Bardo MT Dwoskin LP. Adolescent methylphenidate treatment differentially alters adult impulsivity and hyperactivity in the spontaneously hypertensive rat model of ADHD. Pharmacol Biochem Be. (2016) 141:66–77. doi: 10.1016/j.pbb.2015.12.002, PMID: 26657171 PMC4764879

[53] Sagvolden T Sergeant JA. Attention deficit/hyperactivity disorder—from brain dysfunctions to behaviour. Behav Brain Res. (1998) 94:1–10. doi: 10.1016/s0166-4328(97)00164-2, PMID: 9708834

[54] Sagvolden T Aase H Zeiner P Berger D. Altered reinforcement mechanisms in attention-deficit/hyperactivity disorder. Behav Brain Res. (1998) 94:61–71. doi: 10.1016/s0166-4328(97)00170-8 9708840

[55] Sleator EK Ullmann RK. Can the physician diagnose hyperactivity in the office? Pediatrics. (1981) 67:13–7. doi: 10.1542/peds.67.1.13, PMID: 7243422

[56] Brower VG Fu Y Matta SG Sharp BM. Rat strain differences in nicotine self-administration using an unlimited access paradigm. Brain Res. (2002) 930:12–20. doi: 10.1016/s0006-8993(01)03375-3, PMID: 11879790

[57] Smith TT Levin ME Schassburger RL Buffalari DM Sved AF Donny EC. Gradual and immediate nicotine reduction result in similar low-dose nicotine self-administration. Nicotine Tob Res. (2013) 15:1918–25. doi: 10.1093/ntr/ntt082, PMID: 23817582 PMC3790635

[58] Burke AR McCormick CM Pellis SM Lukkes JL. Impact of adolescent social experiences on behavior and neural circuits implicated in mental illnesses. Neurosci Biobehav Rev. (2017) 76:280–300. doi: 10.1016/j.neubiorev.2017.01.018, PMID: 28111268

[59] Rhodes JD Pelham WE Gnagy EM Shiffman S Derefinko KJ Molina BSG. Cigarette smoking and ADHD: an examination of Prognostically relevant smoking behaviors among adolescents and young adults. Psychol Addict Behav. (2016) 30:588–600. doi: 10.1037/adb0000188, PMID: 27824233 PMC5117481

[60] Wilens TE Biederman J. Alcohol, drugs, and attention-deficit/hyperactivity disorder: a model for the study of addictions in youth. J Psychopharmacol. (2006) 20:580–8. doi: 10.1177/0269881105058776, PMID: 16174669

[61] Blendy JA Strasser A Walters CL Perkins KA Patterson F Berkowitz R. Reduced nicotine reward in obesity: cross-comparison in human and mouse. Psychopharmacology. (2005) 180:306–15. doi: 10.1007/s00213-005-2167-9, PMID: 15719224

[62] Lê AD Li Z Funk D Shram M Li TK Shaham Y. Increased vulnerability to nicotine self-administration and relapse in alcohol-naive offspring of rats selectively bred for high alcohol intake. J Neurosci. (2006) 26:1872–9. doi: 10.1523/jneurosci.4895-05.2006, PMID: 16467536 PMC6793634

[63] O’Dell LE Natividad LA Pipkin JA Roman F Torres I Jurado J. Enhanced nicotine self-administration and suppressed dopaminergic systems in a rat model of diabetes. Addict Biol. (2013) 19:1006–19. doi: 10.1111/adb.12074, PMID: 23834715 PMC3842417

[64] Rupprecht LE Smith TT Donny EC Sved AF. Self-administered nicotine differentially impacts body weight gain in obesity-prone and obesity-resistant rats. Physiol Behav. (2017) 176:71–5. doi: 10.1016/j.physbeh.2017.02.007, PMID: 28189503 PMC6044443

